# Acute chagas outbreaks: molecular and biological features of *Trypanosoma cruzi* isolates, and clinical aspects of acute cases in Santander, Colombia

**DOI:** 10.1186/s13071-015-1218-2

**Published:** 2015-11-26

**Authors:** Martha Lucía Díaz, Sandra Leal, Julio César Mantilla, Alfredo Molina-Berríos, Rodrigo López-Muñoz, Aldo Solari, Patricia Escobar, Clara Isabel González Rugeles

**Affiliations:** Grupo de Inmunología y Epidemiología Molecular (GIEM), Facultad de Salud, Universidad Industrial de Santander, Bucaramanga, Colombia; Grupo de Investigación en Enfermedades Tropicales (CINTROP), Departamento de Ciencias Básicas, Escuela de Medicina, Universidad Industrial de Santander, Bucaramanga, Colombia; Programa de Biología Celular y Molecular, Instituto de Ciencias Biomédicas, Facultad de Medicina, Universidad de Chile, Santiago, Chile; Programa de Farmacología Molecular y Clínica, Instituto de Ciencias Biomédicas, Facultad de Medicina, Universidad de Chile, Santiago, Chile; Present address: Laboratorio de Farmacología y Farmacogenética, Instituto de Investigación en Ciencias Odontológicas (ICOD), Facultad de Odontología, Universidad de Chile, Santiago, Chile; Present address: Instituto de Farmacología y Morfofisiología, Facultad de Ciencias Veterinarias, Universidad Austral de Chile, Valdivia, Chile; Escuela de Microbiología, Facultad de Salud, Carrera 32 #29-31, Oficina 419, Universidad Industrial de Santander, Bucaramanga, Colombia

**Keywords:** *Trypanosoma cruzi*, Acute Chagas disease, Outbreaks, Nifurtimox, Benznidazole, DTU I, Colombia

## Abstract

**Background:**

Outbreaks of acute Chagas disease associated with oral transmission are easily detected nowadays with trained health personnel in areas of low endemicity, or in which the vector transmission has been interrupted. Given the biological and genetic diversity of *Trypanosoma cruzi*, the high morbidity, mortality, and the observed therapeutic failure, new characteristics of these outbreaks need to be addressed at different levels, both in *Trypanosoma cruzi* as in patient response. The aim of this work was to evaluate the patient’s features involved in six outbreaks of acute Chagas disease which occurred in Santander, Colombia, and the characteristics of *Trypanosoma cruzi* clones isolated from these patients, to establish the potential relationship between the etiologic agent features with host behavior.

**Methods:**

The clinical, pathological and epidemiological aspects of outbreaks were analyzed. In addition, *Trypanosoma cruzi* clones were biologically characterized both *in vitro* and in vivo, and the susceptibility to the classical trypanocidal drugs nifurtimox and benznidazole was evaluated. *Trypanosoma cruzi* clones were genotyped by means of mini-exon intergenic spacer and cytochrome b genes sequencing.

**Results:**

All clones were DTU I, and based on the mini-exon intergenic spacer, belong to two genotypes: G2 related with sub-urban, and G11 with rural outbreaks. Girón outbreak clones with higher susceptibility to drugs presented G2 genotype and C/T transition in *Cyt b*. The outbreaks affected mainly young population (±25.9 years), and the mortality rate was 10 %. The cardiac tissue showed intense inflammatory infiltrate, myocardial necrosis and abundant amastigote nests. However, although the gastrointestinal tissue was congestive, no inflammation or parasites were observed.

**Conclusions:**

Although all clones belong to DTU I, two intra-DTU genotypes were found with the sequencing of the mini-exon intergenic spacer, however there is no strict correlation between genetic groups, the cycles of the parasite or the clinical forms of the disease. *Trypanosoma cruzi* clones from Girón with higher sensitivity to nifurtimox presented a particular G2 genotype and C/T transition in *Cyt b*. When the diagnosis was early, the patients responded well to antichagasic treatment, which highlights the importance of diagnosis and treatment early to prevent fatal outcomes associated with these acute episodes.

**Electronic supplementary material:**

The online version of this article (doi:10.1186/s13071-015-1218-2) contains supplementary material, which is available to authorized users.

## Background

Chagas disease (CD) caused by *Trypanosoma cruzi* affects about 8 million people in Latin America [1]. In Colombia, it is estimated that 1.3 million people are infected, and 3.6 million are at risk [2]. The department of Santander has a seroprevalence rate close to 40 % in most endemic areas [2]. CD has two clinical phases: acute phase is usually asymptomatic, and chronic phase in which about 10-30 % of infected patients develop symptoms [3], and 70 % could remain asymptomatic, (indeterminate form) [3]. Chronic Chagasic cardiomyopathy (CCC) is the most common and severe manifestation, and occurs after 10 – 20 years following the infection. The digestive forms of CD occur almost exclusively in Argentina, Brazil, Chile and Bolivia, although they have also been reported in Mexico, and Colombia [3–5].

Differences in biological characteristics among *T. cruzi* isolates have been demostrated [6, 7], and the correlation between genotypic and phenotypic aspects of parasite behaviour was confirmed by Revollo et al. [8] Some of these biological features of the *T. cruzi* genotypes are relevant, since they might be associated with pathogenesis or drug susceptibility [9]. In this regard, efforts to analyze the relevance of these differences in pathogenesis of CD are necessary. *T. cruzi* has a high degree of genetic variability, and is classified in six phylogenetic groups (discrete typing units, DTUs), categorized from TcI to TcVI [10]. In Colombia, most isolates obtained from diverse sources have been classified as TcI, but in the Santander Department, TcII have also been found in chronic cases of CD [11–15]. A new subdivision within TcI parasites has been reported using nuclear and mitochondrial molecular markers as miniexon and cytochrome b gene sequencing, respectively [16–18]. Some of these TcI variants seem to be associated with humans and peridomestic and sylvatic transmission cycles [18]. However, recent reviews identified that although there are genetic and geographical structures, these are not strictly associated with cycle and host origins [19].

No vaccines are available so far, and there are only two registered drugs, the nitrofuran derivative, nifurtimox (Lampit, Bayer) and 2-nitroimidazole benznidazole (Radanil, Roche), being especially effective in newborns, and in the acute phase [1]. However, these drugs have severe limitations of long protocols of treatment and potential harmful side-effects. Also, *T. cruzi* strains with natural resistance have been reported [20], and these drugs have limited efficacy, depending on the phase of infection, the patient’s age, and the involved endemic area. [21, 22] This last point could be related to differences in drug susceptibility among *T. cruzi* genetic variants [1, 20].

In addition to chronic CD, acute cases of CD have been reported early in French Guiana and Brazil since 1941 [23]. Currently, close to 78 % of the acute outbreaks are related with oral transmission through contaminated food [23, 24]. Most of them are reported in the Amazonian region and Southern Brazil, Venezuela, and French Guiana [24–30]. Although, in most of the acute cases TcI parasites were found; TcII, TcIII, TcIV and TcV have also been documented [31–34]. In Colombia, the first acute CD report was in the Norte de Santander Department, in 1992 [35]. Subsequently, in the period 2002–2005, other cases were reported from several geographic regions of Colombia, including Santander Department [36]. In 2006, nine new acute CD cases were reported [37]. Between 2008 and 2009 outbreaks of probable oral transmission were reported in Santander [33, 38]. The purpose of this study was to evaluate the clinical and pathological features of patients involved in six outbreaks from Santander, between 2008 to 2010, and the biological and genetic characteristics of *T. cruzi* clones isolated from twelve of these patients. We report that all clones were DTU I, with intra-DTU genotypes G2 and G11. These genotypes had differential distribution in urban and rural areas, and the G2 genotype was more susceptible to drugs analysed.

## Methods

### Ethics, consent and permissions

Patients were included in the study after written informed consent, according to the declaration of Helsinki. For children enrolled in the study, written informed consent was given by parents. The study was approved by the Ethical Committee of the Universidad Industrial de Santander (approval number 012/2009). Patients diagnosed with CD were treated according to the guidelines of the Colombian Health Ministry.

All animal handling protocols were performed according to the “Guide for the Care and Use of Laboratory Animals”, from the National Institute of Health, USA (National Research Council (US) Committee for the Update of the Guide for the Care and Use of Laboratory Animals. Guide for the care and use of laboratory animals (8th edition), and approved by the Institutional Ethical Committee at the Faculty of Medicine, University of Chile (Protocol CBA# 0448 FMUCH).

### Study areas

**Fig. 1:**
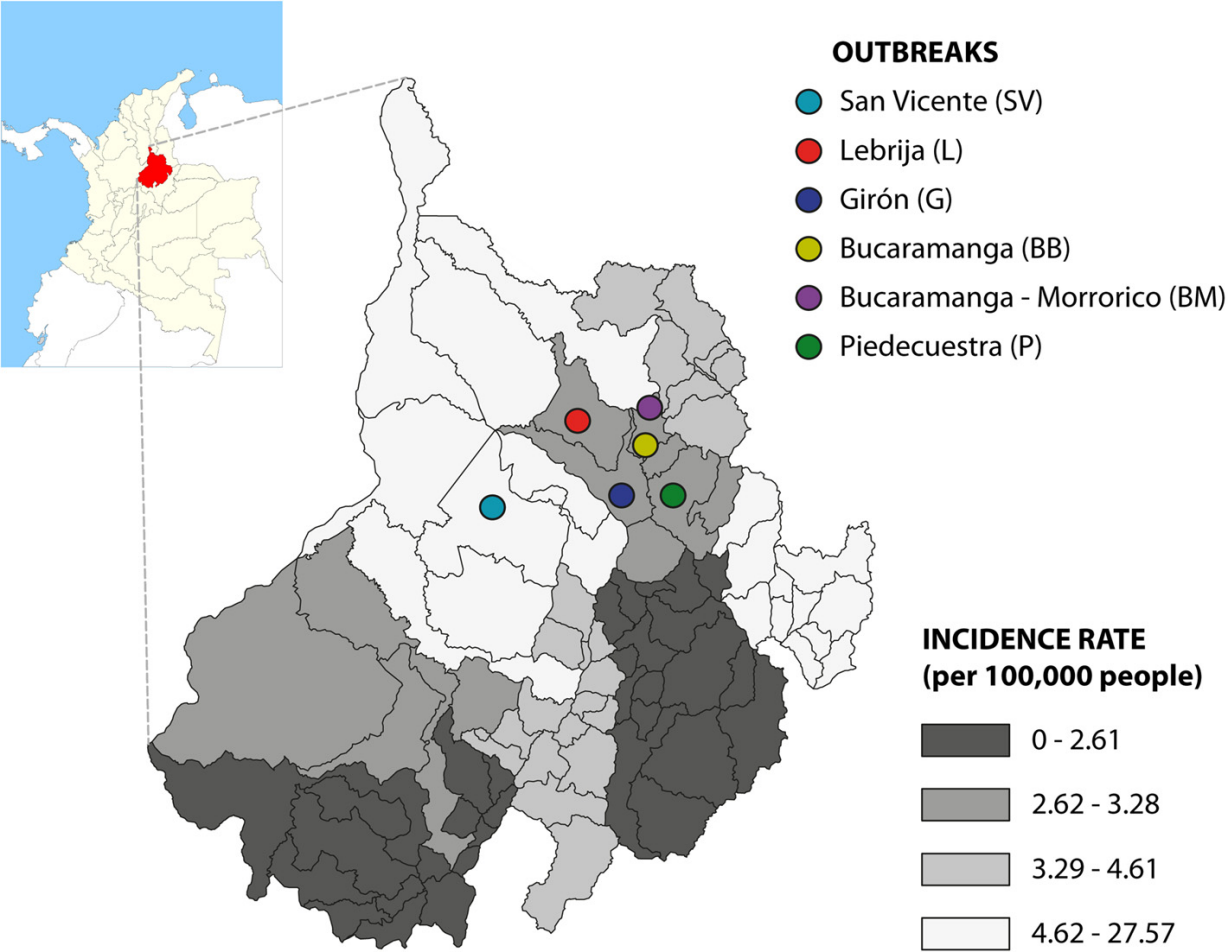
Geographical distribution and location of the municipalities in Santander, Colombia where acute Chagas outbreaks occurred. Gray-scaled map shows the Santander department. The Chagas Disease incidence in each municipality is indicated with one of the four gray scales indicated in the picture. The geographical distribution of the outbreaks described in this article is specified with the colored circles

Six outbreaks of acute CD were reported in five localities of the Santander Department. Santander is one of the 32 departments of Colombia located in the northeastern part of the country. The area is 30,537 km^2^ with a population of 2’086.649 inhabitants, 87 municipalities and Bucaramanga as its capital. Santander Department is considered the third most endemic department for CD in Colombia [39]. According to the Santander Health authorities, six municipalities are considered highly endemic, some of which had improvement of rural housing and vector control program [40]. Municipalities where outbreaks occurred are considered of low endemicity (incidence rates below 3 000 cases per 100 000 inhabitants), except Lebrija and San Vicente de Chucurí with an incidence rate of 12 to 24000 cases per 100 000 inhabitants, and where there are no reports of domiciliated vectors. The first outbreak occurred in December 2008 in the town of Lebrija, in 2009 there were two outbreaks, one in a peri-urban area of the Bucaramanga city (Barrio Bucaramanga), and another in Piedecuesta. In 2010 three outbreaks occurred, one in the town of San Vicente de Chucurí, another in the town of Girón, and the third in a peri-urban area of Bucaramanga city (Barrio Morrorico) (Fig. 1).

### Parasites

Twelve *T. cruzi* isolates from 30 patients of six outbreaks of acute CD (2008 to 2010) in Santander Department from Colombia were used in this study (Table 1). In addition, the 338clBb (TcI) and Sylvio X10 (TcI) were used as a chronic isolate and reference strain, respectively (non-outbreak strains). The parasites were isolated by hemocultures as previously described [41]. The isolates were cloned using an isolation method of single colonies on solid medium [42], resulting in 5–10 clones per isolate, for each isolate one clone was selected. The selected clones from the different outbreaks were maintained in LIT medium and subsequently submitted to the different analysis. The parasites codes, date and place of collection, and gender and age of the patients are showed in Table 1.

**Table 1:** *Trypanosoma cruzi* isolates from acute Chagas disease in Santander, Colombia

ID isolate	Outbreak	Year	Gender	Age (years)
MHOM/CO/08/AM^a^	Lebrija (L)	2008	M	23
MHOM/CO/08/JCR^a^	Lebrija(L)	2008	M	21
MHOM/CO/08/EH	Lebrija (L)	2008	M	22
MHOM/CO/09/JCHV^a^	Barrio Bucaramanga (BB)	2009	F	3
MHOM/CO/09/PEGAL	Piedecuesta (P)	2009	M	6
MHOM/CO/10/SMAY	San Vicente (SV)	2010	F	24
MHOM/CO/10/GUICA	San Vicente (SV)	2010	F	53
MHOM/CO/10/SATHE	Girón (G)	2010	F	8
MHOM/CO/10/DATHE	Girón (G)	2010	F	3
MHOM/CO/10/OSGO	Girón (G)	2010	M	19
MHOM/CO/10/JLM	Girón (G)	2010	M	19
MHOM/CO/10/HEMA	Morrorico Bucaramanga (MB)	2010	M	43

^a^Fatal cases

### Genotyping of *T. cruzi* isolates

Seven independent genetic markers were used to genotype the *T. cruzi* clones. Mini-exon intergenic spacer (SL-IR), 24Sα rRNA D7 domain and 18S rRNA, were amplified as previously described [43, 44]. The mitochondrial gene Cytochrome Oxidase subunit II (COII) was characterized by nested PCR followed by Alu I restriction endonuclease. The RFLP analysis was done in 6.0 % polyacrylamide gel and revealed by silver staining [45], and the Cytochrome b (*Cyt b*) gen was amplified as previously described [46]. Additionally, HSP60 and GPI genes were analysed [47]. DNA from *T. cruzi* strains, representing major genetic groups TcI and TcII were used as controls for all PCR assays. In addition, to determine the degree of genetic similarity of the parasites with the sequences already reported in GenBank, mini-exon PCR products were sequenced from *T. cruzi* clones in the ABI 3730xl DNA analyzer (Applied Biosystems, Foster City, CA, USA). Purification and direct sequencing of DNA amplicons of *Cytb* gene were performed by MACROGEN (Seoul, South Korea).

### Editing and alignment

SL-IR sequences and *Cyt b* genes were edited using BioEdit software v.7.0.9 [48], and aligned with ClustalW algorithm [49]. For SL-IR the sequence was resolved for 192 positions taking into account the microsatellite region at the beginning of the sequences and with the putative motif (GT)n (ATGT)n (AT)n (GT)n and the end with GCGTGT [19], and for *Cytb* the sequence was resolved for 516 bp. Reference genotypes were obtained from the GenBank database: AM259467 and AM259479.1. The sequences were analysed with the entire microsatellite region and without this motif as was described [19].

### Biological features of *T. cruzi* clones *in vitro*

#### Differentiation of *T. cruzi* epimastigotes

*T. cruzi* epimastigotes (1 × 10^6^parasites/mL) were cultured in Grace’s Insect Media (Sigma, St. Louis, USA) with 10 % hiFCS at 28 °C. Both, numbers of epimastigotes and culture medium-derived trypomastigote (cmDT) were counted under a microscope using a hemocytometer chamber after 2, 5, 7, 10, 12, 14, 17, 19, and 21 days. Epimastigotes with flagellum and which were mobile were counted. Determination of day of maximum cmDT, and the % of cmDT (E + T) were calculated at each point of time.

### Release-kinetic of mammalian cells derived trypomastigotes (cellDT)

Vero cells in RPMI 1640 (Gibco, Grand Island, USA) culture medium supplemented with 10 % hiFCS were allowed to attach to 24-well plates. After 24 h, a final number of 1 × 10^5^ adherent cells/mL were infected with 5 × 10^5^ cmDT/mL at 37 ° C, 5 % CO_2_ for 24 h. Free parasites were removed by washing twice with culture medium. After 24 h, released and mobile cellDT were counted microscopically every day for 15 days using eosin yellow.

### Infectivity to cultured mammalian cells

Vero cells (ATCC: CCL-81), cultured in RPMI medium (10 % hiFCS at 37 °C, 5 % CO_2_ and 95 % humidity), were plated on glass coverslips in 24-well plates and infected with cellDT at a 10:1 parasite-to-cell ratio for 24 h. Non-internalized parasites were removed by washing with culture medium. Infected cultures were incubated for 24, 48 and 72 h. The cells were fixed with 70 % methanol and stained with Giemsa and the percentage of infected cells and the numbers of amastigotes per cell were calculated [25, 26].

### Drug susceptibility test

For amastigote drug susceptibility test, Vero cells were infected with cellDT as described above. Infected cells were treated with benznidazole or nifurtimox ranging from 0.3 to 300 μM for 120 h. The treatment schedule included a second dose after 72 h. Control untreated cells were maintained with culture medium without drug. After 120 h, the cultures were fixed and stained with Giemsa. The percentage of infected cells was evaluated by counting 300 Vero cells per coverslip for each condition by light microscopy. Drug activity was determined by the percentage of infected cells in treated and untreated cultures. The percent of infected cells before the start of the drug treatment was determined in all strains.

### *In vivo* behavior of *T. cruzi* clones

#### Mice infection

BALB/c mice (*n* = 6) of 20–25 g were inoculated orally with 10^6^ cmDT of each *T. cruzi* clones studied from the mortal acute cases (JCR, and JChV), and control clones for comparative purposes 338clBb and Sylvio X10). Parasitemia was registered to assure infection after the 4^th^ day of inoculation. End point for cardiac studies was established at the 25^th^ day post infection.

### Heart extraction and histopathological studies from infected mice

Surviving mice were euthanized at day 25 p.i. and their hearts were extracted. Hearts were longitudinally sectioned to further analysis by histopathology.

### qPCR of heart tissue from infected mice

Heart samples extracted from euthanized animals were homogenized, and the total genomic DNA was isolated using a Wizard® Genomic DNA Purification Kit (Promega, Madison, USA) following the manufacturer’s instructions. A Real Time PCR TaqMan assay was used to quantify parasite DNA with method previously described [50]. The data are expressed as the ratio of *T. cruzi* DNA to murine DNA.

### Statistical analysis

The *in vitro* growth kinetics, metacyclogenesis, cell infectivity, and drug activities experiments were performed in triplicate, with at least two independent experiments. For all experiments, analysis of variance with Bonferroni post-test and *T* test were made, with a confidence level of 95 %, using the Graphpad Prism software version 5.0. Values of *p* <0.05 were considered significant. The drug activities of benznidazole and nifurtimox were expressed as the drug concentration able to decrease in a 50 % (IC_50_) the number of infected cells. The IC_50_ values were determined by four-parameter dose response curve fitting, using the Xlfit4TM program (ID Business Solution, Guildford, UK).

## Results

### Epidemiological, clinical and pathological findings

The highest percentage of cases came from the town of Lebrija (33.3 %), 23.3 % from Bucaramanga, 16.6 % from Piedecuesta and Girón and 10 % from San Vicente de Chucurí. Three outbreaks were from rural areas and three were peri-urban areas. One important epidemiological feature in five of the outbreaks was the presence of marsupial and insects in the peridomiciliary and absence of domiciliated triatomines.

The average age of the patients was 25.9 years and 54 % were male and 46 % were female. The most common symptoms were fever and malaise (100 %), abdominal pain (83.3 %) and tachycardia (75 %). Heart failure was present in 66.6 % and pericardial effusion in 41.6 % of cases (Table 2). The mortality rate was 10 % (3/30) and one patient required a heart transplant to save her.

**Table 2:** Signs and symptoms of patients with acute Chagas disease from Santander, Colombia

Signs and symptoms	N (%)
Fever	12 (100)
Malaise	12 (100)
Abdominal pain	10 (83.3)
Tachycardia	9 (75)
Facial edema	8 (66.6)
Headache	8 (66.6)
Heart failure	8 (66.6)
Nausea	7 (58.3)
Breathing difficulty	6 (50)
Chest pain	6 (50)
Vomits	5 (41.6)
Legs edema	5 (41.6)
Pericardial effusion	5 (41.6)
Cough	4 (33.3)

### Lebrija outbreak

This outbreak involved 10 individuals. The first patient diagnosed, three family members, two colleagues and four workers of the parents’farm. All patients shared food in this farm, four months before diagnosis. The diagnosis of acute CD was performed by autopsy of the first patient and the other patients were diagnosed by laboratory tests, (parasitological and/or serological tests). All patients were treated with nifurtimox, nevertheless, one of the co-workers of the first patient also died as a consequence of heart failure. The parent’s house was built with wall of brick with a roof made of tile and cane, and the floor is made of cement. The kitchen zone was located in the back part of the house, it was observed that there were no access to animals and there were no windows. Three *T. cruzi* isolates were obtained from these patients.

**Fig. 2:**
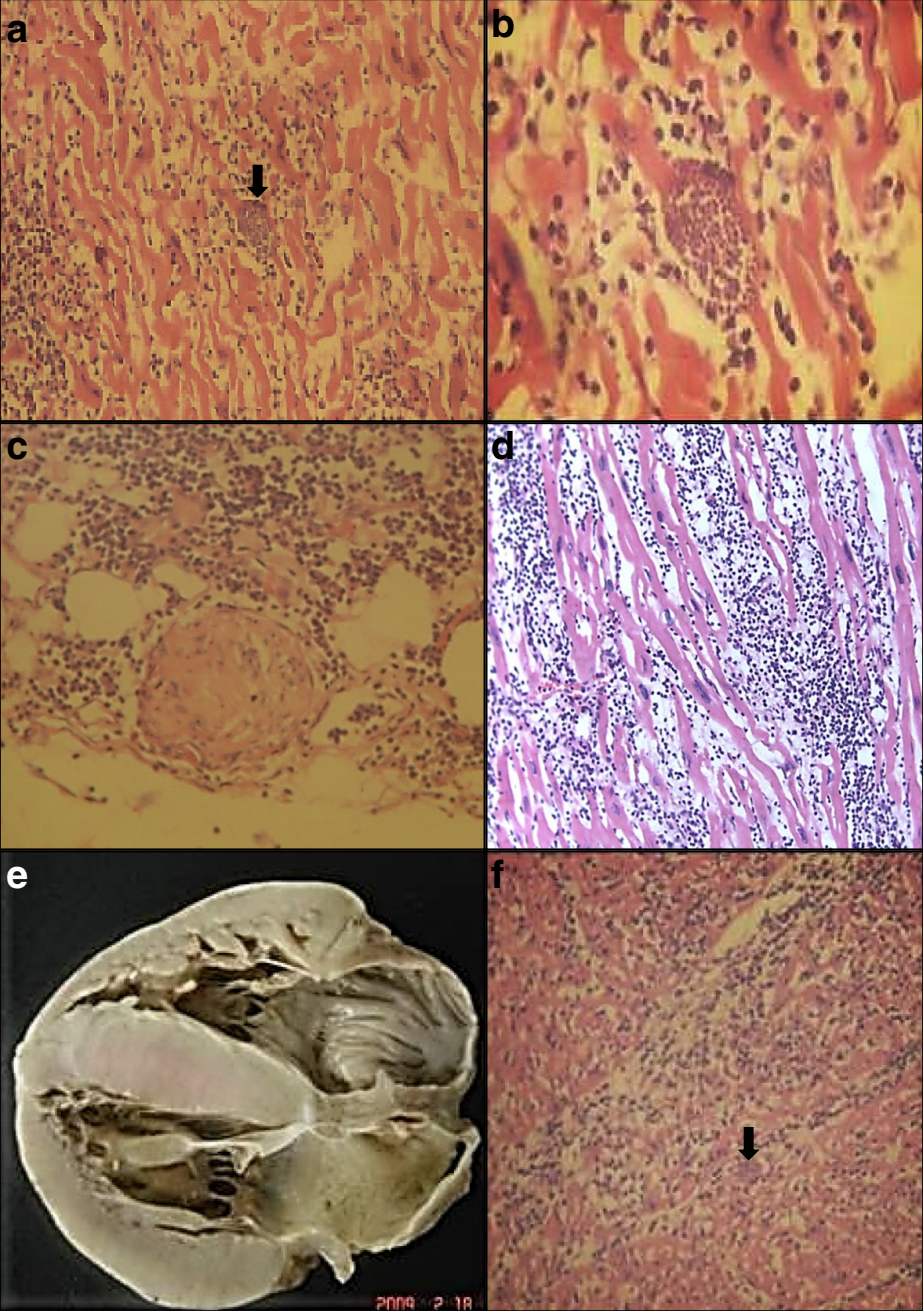
Histopathology analysis of hearts from acute Chagas cases occurred in Santander, Colombia. Figures **a**, and **b**. Heart sections of the patient 1 from Lebrija outbreak. **c**. Heart section of the patient 2 from Lebrija outbreak. **d**. Heart section of the patient from San Vicente outbreak. **e**. Overview of the heart of the patient from Bucaramanga (BB) outbreak. **f**. Heart section of the same patient showed in panel **e**. (Bucaramanga outbreak). Amastigote nests are indicated by black arrows

In summary, in this outbreak two fatal cases occurred. The case 1 was diagnosed post-mortem, and whose findings were soft tissue edema, bilateral hydrothorax, ascites, pulmonary edema and cardiomegaly with dilatation of all cavities. Histological examination of the heart showed dense inflammatory infiltrate with diffuse involvement of the myocardium, abundant cysts occupying most of the myocardial fibers, *T. cruzi* amastigote occurrence, and extensive areas of myocardial necrosis (Fig. 2a, b). At the level of the esophagus and stomach congestion was observed marked with lymphocytic infiltrate in the absence of parasites.

The second case was a patient diagnosed by serological tests; this patient was treated with nifurtimox, with satisfactory evolution. A week following discharge from the hospital, the patient was readmitted with dyspnea, orthopnea, cough, hemoptysis but no fever or pain. Clinical evaluation revealed evident arrhythmic heart sounds, decreased breath sounds, and hepatomegaly. The electrocardiogram revealed atrial fibrillation with high ventricular response, and chest radiograph evidenced right pleural effusion. He was moved to the intensive care unit with congestive heart failure diagnosis, and there, the patient had progressive deterioration of cardiocirculatory function and developed cardiogenic shock refractory to the administration of intravenous fluids and inotropic drugs, and he died. The autopsy showed predominantly mononuclear inflammatory infiltrate of lymphocytes in the epicardium and neural ganglia, extending diffusely throughout the myocardium. Numerous changes were also observed in the myocardial fibers with necrosis and scarce amastigote nests (Fig. 2c). Esophagus, small intestine and colon showed marked congestion without inflammatory disorder, or parasites.

### Bucaramanga outbreak

The Bucaramanga outbreak occurred in a peri-urban area of Bucaramanga city in February 2009 and involved five cases of one family: a one year old girl who died, her mother, her sister, her aunt, and her uncle. The diagnosis of acute CD was performed on autopsy of the index case and the other patients were diagnosed by laboratory tests, (parasitological and/or serological tests). The cases had symptoms about 15 days before the girl death. All patients were treated with nifurtimox. The house is located in an area surrounded by a wooded area with the presence of wild animals: rats, marsupials and monkeys. 63 % of the neighbors recognized the insect vector, 23 % reported having seen them in the house, in bedroom walls, ceilings and flying out of the house (17 %) [38]. One *T. cruzi* isolate was obtained. The girl was diagnosed by autopsy whose findings were: generalized edema, bilateral pleural effusions and pericardial ascites, cardiomegaly (Fig. 2e), linfoplasmohistiocitaria global severe myocarditis by *T. cruzi* and amastigote nests (Fig. 2f). Pulmonary edema, hepatosplenomegaly and generalized mesenteric lymphadenitis were also observed.

### Piedecuesta outbreak

In April of 2009 in a rural area of the Piedecuesta locality occurred the third outbreak which involved five cases of one family: one boy of six years old, the mother, the grandmother, a sister and one uncle. The diagnosis was made by clinical symptoms and confirmed by parasitological (boy) and serological tests (family). The house is located in an area surrounded by a wooded area. The kitchen was located at the back of the house and was easily accessible from the outside. Near the house was a deposit which generated dirt and proliferation of insect vectors and different animals, however, at home and nearby no triatomines were found. All patients were treated with nifurtimox and they recovered. One *T. cruzi* isolate was obtained.

### San Vicente de chucurí outbreak

In a rural area of the San Vicente de Chucurí locality in February 2010 occurred an outbreak involving three cases: one woman of twenty four years old whose diagnosis was made by parasitological test, and two other cases: the woman’s mother and a colleague who were identified by serological test. The house was located in the vicinity of the town, built with walls, roofs and floors with solid materials and was surrounded by grasslands, palm trees and a wooded area where insect vectors and marsupials were found. The patients were treated with benznidazole. Two *T. cruzi* isolates were obtained.

The young woman had a positive hemoculture, and clinically presented chest pain, tachycardia, pericardial effusion, evolving to heart failure. Therefore, the patient was subjected to heart transplantation to save her. In the pathological analysis, the infected heart was normal size and shape, with 355 g of weight. It presented little lymphocytic infiltration in the epicardium and the myocardium presented severe necro-inflammatory lesions represented by a dense infiltrate of lymphocytes, plasma cells and histiocytes and multiple areas of necrosis with multiple nests occupying almost the entire cytoplasm of some myocardial fibers, in which punctate structures were found inside with diameter between 1–2 microns, which corresponded to *T. cruzi* amastigotes. The coronary arteries had normal characteristics and no plaques were identified (Fig. 2d).

### Girón outbreak

In May of 2010 in a peri-urban area of the Girón locality there was an outbreak involving five cases in one family: one man of 19 years old who had symptoms 15 days after the diagnosis was made by parasitological and serological test. The other cases were: two sisters, a young man, and a mother. All patients shared a meal and all were treated with benznidazole. The house was made of brick and cement floor, but near to the house were palm trees with the presence of triatomines. Four *T. cruzi* isolates were obtained.

### Morrorico outbreak (Bucaramanga)

In the Morrorico district, a peri-urban area of Bucaramanga city (BM), in June of 2010 there was an outbreak with two cases: one man (index case) and his wife. The diagnosis of the index case was made by parasitological and serological test and the main symptoms were fever, headache, malaise, facial edema, tachycardia and heart failure. His wife had fever and malaise. The kitchen of the house was open to the yard that is very close to fruit trees and a wooded area. From the survey it was known that the patient hunted animals like armadillos and opossums for food. The patients were treated with benznidazole. One *T. cruzi* isolate was obtained.

### Genotyping of *T. cruzi* isolates

**Fig. 3:**
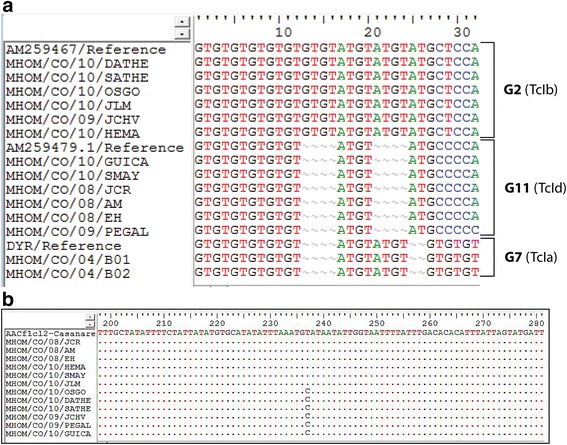
Sequence alignment of the SL-IR microsatellite region, and TcI sub-classification of *T. cruzi* clones obtained of patients with acute Chagas disease in Santander, Colombia. **a**. Alignment of the SL-IR microsatellite region and TcI sub classification. *T. cruzi*isolates and reference strains **b**. Alignment of the *Cyt b*

Genetic characterization with seven different markers allowed us to identify that all the *T. cruzi* isolates of acute cases belong to DTU I (Additional file 1: Figure S1). Sequencing and alignments of SL-IR showed that *T. cruzi* clones were grouped into two distinct genotypes: G2 (50 %) with the putative motif in microsatellite (GT)_8_, (ATGT)_2_, (AT)_1_ and (GT)_0_^19^ or Tc Ib previously described [17] for clones of Girón, Barrio Bucaramanga and Morrorico; and G11 (50 %) with the putative motif in microsatellite (GT)_6_, (ATGT)_1_, (AT)_1_ and (GT)_0_ or Tc Id for clones from Lebrija, San Vicente, and Piedecuesta (Fig. 3a). The analysis of 192 nucleotides without microsatellite region showed 11 variable sites and 181 conserved sites. Although, the alignment of the sequences of *Cyt b* gene showed similarity of all clones with genotype C previously described [51, 52], three clones of Girón, Barrio Bucaramanga, Piedecuesta and one of San Vicente exhibited a C/T transition at position 237 (50 %) unreported previously (Fig. 3b).

### Biological features of *T. cruzi* clones *in vitro*

#### Differentiation of *T. cruzi* epimastigotes

**Fig. 4:**
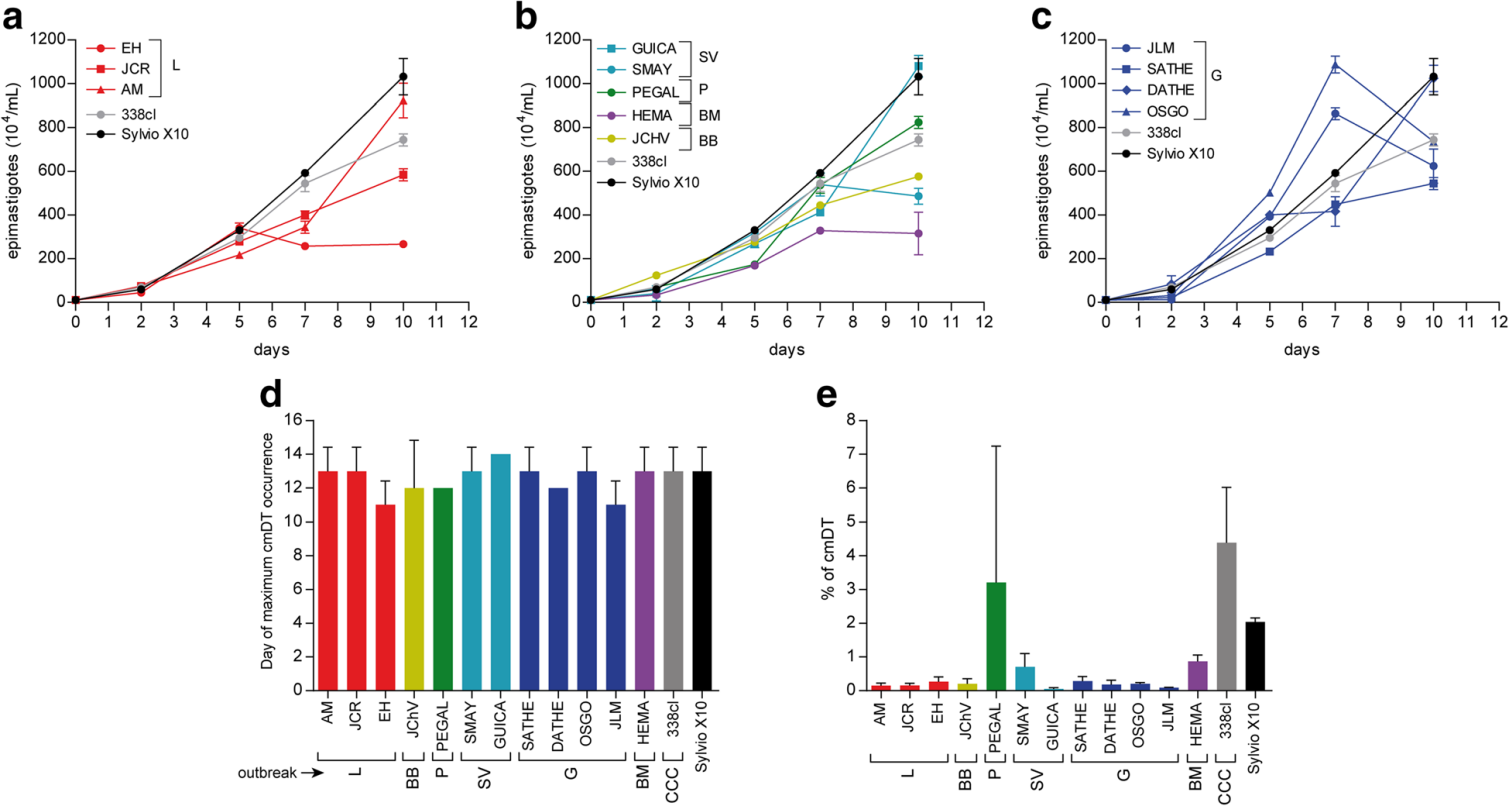
Differentiation of *T. cruzi* epimastigotes. Epimastigotes were cultured in Grace’s medium and the number of epimastigotes and culture medium-derived trypomastigote (cmDT) were evaluated. Figures **a**, **b**, and **c**. Epimastigote numbers after 2, 5, 7, 10 and 12 days of assay. **d**. Day of maximum numbers of cmDT. **e**. Maximum percentage of cmDT in total parasites (epimastigotes plus cmDT). Each experiment was evaluated in triplicate and repeated two times

Kinetics of epimastigote growth between outbreak isolated parasites and non-outbreak (338clBb and Silvio X10) strains are showed in Fig. 4a, b and c. Sylvio X10 strain grows faster than the parasites isolated from Lebrija patients (EH, JCR, and AM, Fig. 4a) and also from Morrorico, Barrio Bucaramanga and San Vicente patients (Fig. 4b). Parasites isolated from Girón patients (Fig. 4c), showed different patterns of growth: OSGO and JLM grow faster than the others showing the maximum parasite numbers at 7 days, SATHE is the slowest one, and DATHE showed similar pattern of growth than Sylvio X10. The non-outbreak isolates used in this study showed a maximal number of epimastigotes (74–103 × 10^6^ parasites/mL) at 10 days (Fig. 4).

For metacyclogenesis in axenic cultures, cmDT reached a maximum between 10 and 15 days, without significant difference between all clones (Fig. 4d). The numbers of cmDT in acute clones compared with reference strains were different (*p* < 0.05). Transformation values below 1 % on outbreak clones compared with 4.5 and 2 % on 338clBb and Sylvio X-10 clones respectively were observed. The only exception among the acute clones was the Piedecuesta clone that reached a 3 % of cmDT transformation (Fig. 4e).

### Parasites behavior in mammalian cell culture

**Fig. 5:**
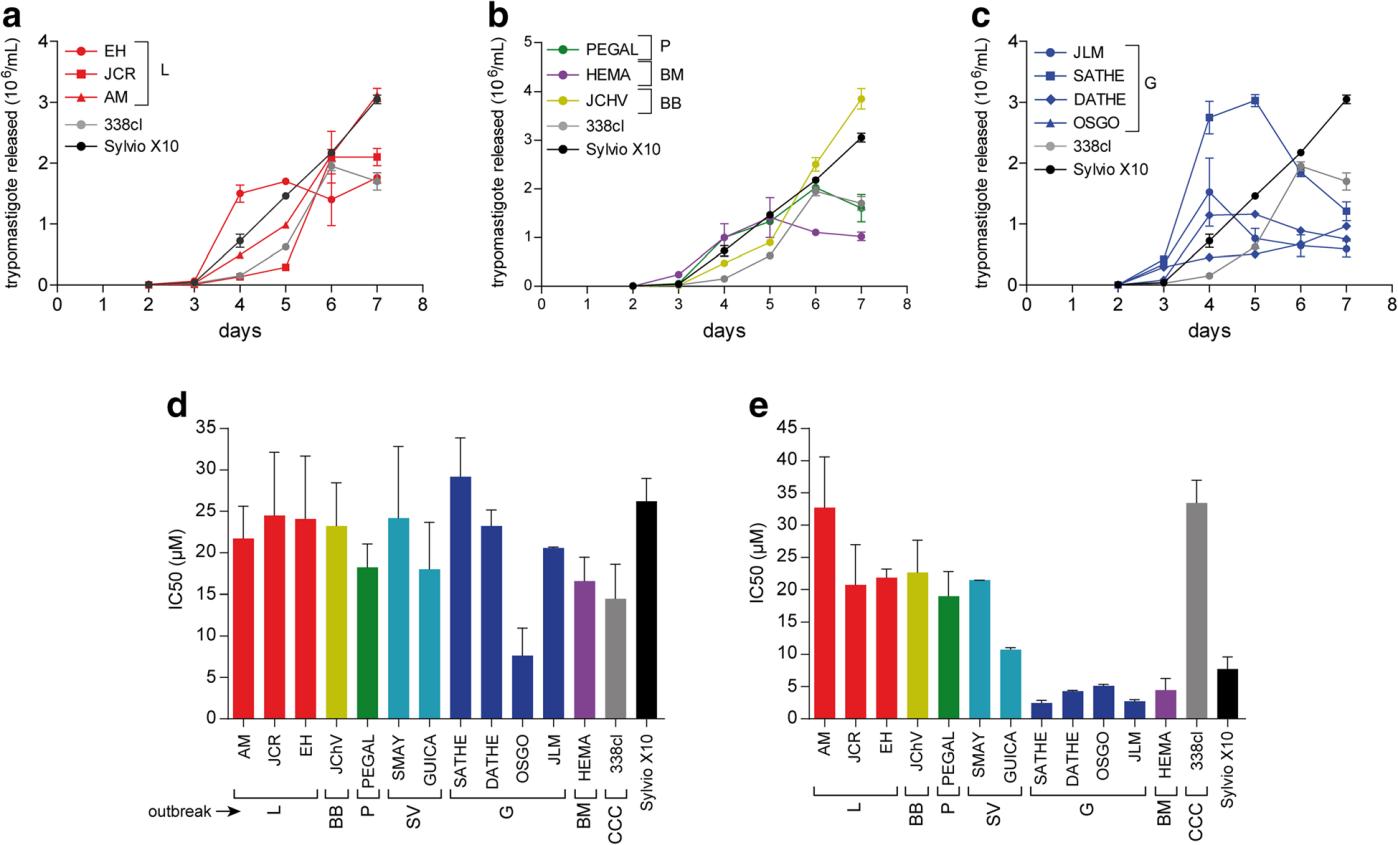
Release of cell derived tripomastigotes (cellDT) and drug susceptibilities of acute outbreaks-parasites. Figures **a**, **b** and **c**. Epimastigotes were allowed to infect VERO cells and released cellDT were counted each day. The figures showed the numbers of cellDT released by each clone. Figures **d** and **e**. Vero cells were infected with cellDT and treated with increased concentrations of benznidazole (BNZ, figure **d**) and nifurtimox (NFZ, figure **e**) for 5 days. Each drug concentration was evaluated in triplicate and the experiment was repeated two times. The percent of infected cells before the experiment was 60–90 %

All *T. cruzi* clones were able to infect Vero cells and transform to amastigotes, showing an increased number of intracellular parasites up to 72 h (data not shown). A slight increase of the per cent of infected cells in all outbreak clones after infection was registered with values in the range of 30–48 % at 24 h, 33–70 % at 48 h and 43–74 % at 72 h. A statistically significant (*p* > 0.05) higher degree of cell infectivity was observed in the reference Sylvio X10 strain with values of 92.3 % at 24 h, 93.3 % at 48 h and 96.6 % at 72 h. All the tested clones produced cellDT from Vero cells as is showed in Fig. 5a, b and c. JLM and SATHE (both Giron clones) and EH (Lebrija clone) reached the maximum number of cellDT earlier than Sylvio X10 clone and JCHV (Barrio Bucaramanga clone) produced a greater number of cellDT than Sylvio X10 clone.

### Drug susceptibility

We also studied the susceptibility of each clone to nifurtimox and benznidazole. At IC_50_ level, the ranges of susceptibilities were from 2.15 ± 0.17 to 38.28 ± 1.70 μM to nifurtimox, and 5.30 ± 0.46 to 32.50 ± 3.72 μM to benznidazole. Most of the clones were more susceptible to nifurtimox than benznidazole, *p* < 0.05 (Fig. 5d and e). Overall, the most sensitive clones to nifurtimox were those belonging to the Girón outbreak: SATHE, DATHE, OSGO and JLM and Morrorico clone (HEMA), these clones showed a similar susceptibility pattern as the Sylvio X10 reference clone (Fig. 5d).

### *In vivo* behavior of clones

**Fig. 6:**
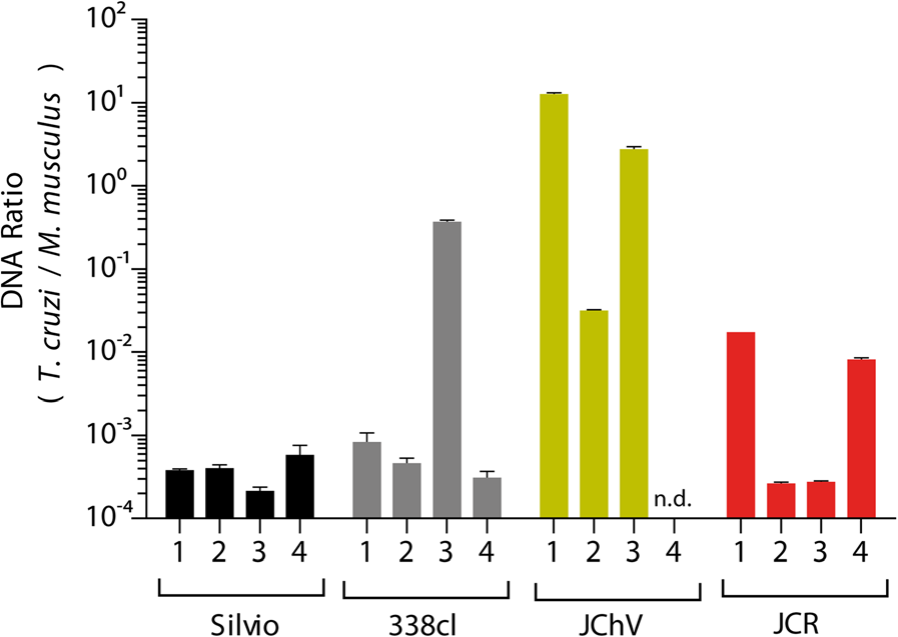
Parasite load quantitation in heart from mice infected orally with *T. cruzi* clones. BALB/c mice were infected with four different clones and the parasite load was measured by qPCR at day 20 post-infection. Each bar represents one mouse measured by triplicate

To explore the possibility of an oral infection in our previous cases, we studied the infectivity by oral route of two clones from lethal cases (JChV and JCR clones). Also, we used the 338clBb clone, obtained from a chronic case, as a control. 10^6^cmDT from the JChV, JCR and 338clBb clones were inoculated by oral route in BALB/c mice and the outcome of the infection was compared with the Sylvio X10 clone, inoculated by the same route. All clones inoculated by the oral route successfully infected BALB/c mice, which was corroborated by parasitemia analysis (data not shown). Only the JChV clone showed mortality at day 25 post infection (1 of 4 mice). The cardiac outcome of the infection was studied by histology (Table 3) and parasite load in cardiac tissue by qPCR (Fig. 6). As Table 3 shows, JChV-infected mice presented more inflammatory infiltrate and amastigote nests. This correlates with the qPCR quantification, where mice infected with JChV clone had 10^4^ more parasites than mice infected with Sylvio X 10 clone, used as reference. In addition, 66 % (2 of 3) mice infected with the JChV clone showed one or more amastigote nests in the histopathology. JCR and 338clBb clones induced low inflammation in cardiac tissue, similar to Sylvio X10 infection. JCR-infected mice did not show amastigote nests, whereas 50 % (2 of 4) of mice infected with 338clBb clone did, but to a lesser extent than the JChV infection (Fig. 6).

**Table 3:** Histopathology of tissues from mice with acute Chagas disease infected orally with experimental and control clones

ID isolate	Numbering of mice^a^	Inflammatory infiltrate	Amastigote nests^b^
MHOM/CO/09/JCHV	1	Moderate	(0-1/c 40X)
	2	Moderate	(2-3/c 40X)
	3	Abundant	None
MHOM/CO/08/JCR	1	Scarce	None
	2	Scarce	None
	3	None	None
	4	Scarce	None
MHOM/CO/01/338clBb	1	Scarce	None
	2	Scarce	None
	3	Scarce	(0-1/c 40X)
	4	Scarce	(0-1/c 40X)
Sylvio X10	1	Scarce	None
	2	None	None
	3	Scarce	None
	4	Scarce	None

^a^Numbering mice correlate with those shown in Fig. 6 (qPCR)

^b^No. of amastigote nests found on examination 40X

## Discussion

In Colombia, the departments with the highest infestation prevalence are located in the northeastern region [39], and since 1996 with the start of the vector control programs the number of domiciliated vectors decreased in highly endemic regions [53]. However, risk for human infection continue due to the natural infection rates in most sylvatic triatomine species that sporadically invade human dwellings, and the presence of houses close to palm trees occupied by triatomines and marsupials, both frequently infected [54, 55]. The mammalian reservoirs infected as well as the massive displacement of human populations have increased the risk of transmission of *T. cruzi* even in areas of low endemicity. In Colombia, similar to what happened in northeast Brazil and Venezuela, these factors would be related to the increased outbreak reports of acute CD probably associated with oral transmission by food contamination [29, 32].

Santander has been the department with the highest number of outbreaks described (6/9). Although it is the third department of Colombia with triatomine house infestation [39], most of the outbreaks occurred in towns of low endemicity in which there are no reports of domiciliated triatomines. This finding and the fact that all cases occurred in rural or suburban areas with conditions favoring entry of sylvatic vectors or wild reservoirs to housing, support the hypothesis of oral transmission [29]. There is also the possibility of transmission by food contamination with urine or anal secretions of infected marsupials [55, 56]. In five of the outbreaks of this study, the presence of marsupials in the peridomestic area was reported.

These outbreaks involved a small number of infected individuals, primarily associated with family outbreaks or individuals who attended and fed in the same house. The mortality was high especially during the first outbreak, probably due to late diagnosis by nonspecific clinical symptoms and because these cases were unusual in the region. The clinical suspicion is directed to other endemic diseases particularly dengue with similar early clinical symptoms. The frequency and the symptoms observed were similar to those in the majority of acute CD outbreaks reported [25, 27, 29, 32, 57, 58]. The histopathological findings of four hearts from patients, showed an intense inflammatory infiltrate in the interstitium, and the presence of amastigote nests in myocardial fibers. However, this inflammatory injury not only compromised the myocardial fibers but also damaged the ganglion cells and nerve fibers of the heart. These results contrast with chronic forms in which there is prevalence of fibrosis represented by fibroblasts and fibrocytes accompanied by matrix collagen deposit which replaced the myocardial fibers [15]. In digestive tract tissue of three autopsies performed, we observed marked congestion without tissue damage or the presence of parasites. The difference observed between the two patients of the Lebrija outbreak could be related to the fact that case 1 was not treated, and this correlates with the presence of an intense inflammatory infiltrate and numerous amastigote nests. Conversely, the second case that received treatment, also showed abundant inflammatory infiltrate, however, they presented nerve fiber damage and fewer groups of amastigotes. Damage to the nerve fiber generated conduction disturbances, which finally led to heart failure.

The isolates obtained in the six outbreaks belong to TcI that is the DTU most abundant and widely distributed in America, both in the domestic and sylvatic environments. In Colombia this DTU is dominant and it is associated with CCC [11, 12, 14]. Based on intergenic sequences of the mini-exon gene the clones were grouped into the G2 and G11 genotypes, which are also the most common in Colombia associated, not only with humans but also with vectors and reservoirs [18, 19]. All clones in each outbreak were genetically identical; however, it is important to clarify that we only analysed one clone of each isolate; therefore we could not confirm the presence of multiclonal infections as has been described in Colombia and Venezuela [33, 59]. Herrera et al, reanalysing the variability of the miniexon microsatellite region of molecular clones from 11 Latin American countries concluded, that although this region showed one interesting TcI variability that allowed some genetic and geographic structure; this structure was not associated with cycle and host origins [19], as previously proposed [14, 17]. However it is noteworthy that in our study the clones grouped as G2 (TcIb) occurred in suburbs of the city surrounded by forested areas with the presence of vectors, and reservoirs of marsupials or other rodents around houses, and the clones identified as G11 (TcId) occurred in rural dwellings suggesting both a sylvatic transmission. These results and recent reports from Venezuela [59], and Colombia [33] of the presence of G2, G7 and G11 genotypes and multiclonal infections in acute CD patients support the notion that even though there is a grouping of genotypes, there is not strict correlation between genetic groups, and the cycles of the parasite or the clinical forms of the disease.

Although the majority of clones had similar biological features related with growth, metacyclogenesis and infectivity of mammalian cells, some differences were observed in Girón outbreak clones which reached maximum growth peaks and numbers of cellDT earlier than reference clone. These clones presented a particular G2 genotype and C/T transition in *Cyt b*. In the in vivo model of experimental infection, we show that oral inoculated *T. cruzi* were able to infect, and further reach the heart, inducing different clinical cardiac outcomes, similar to intraperitoneal infection (data not shown). JChV was the most infective clone; indeed, was the only lethal clone in Balb/c mice (one of four infected mice). This clone produced a greater number of cellDT than reference clone (Sylvio X10). This finding might be related to the fact that this isolate came from a fatal case.

*T. cruzi* clones obtained from all outbreaks were susceptible to both nitroheretocyclic drug *in vitro*, but nifurtimox was more active and showed more heterogeneity in the response than benznidazole. All clones from Girón outbreak, HEMA (Morrorico) and GUICA (San Vicente) showed increased susceptibility to nifurtimox compared with clones of other outbreaks. On the other hand, OSGO (Girón) also was the most susceptible to benznidazole. In a previous study with isolates from humans and vectors of Santander, similar results were obtained related to increased susceptibility to nifurtimox compared with benznidazole [22]. A study analysing acute outbreaks in Venezuela also showed that they were more susceptible to nifurtimox [60]. However, some studies of clinical follow-up of response to treatment with benznidazole of patients with acute CD of outbreaks from Venezuela and Brazil reported the presence of parasites on blood cultures post-therapy indicating treatment failure [34, 61]. In contrast, in our work, the drug susceptibility *in vitro* was correlated with the response to treatment with benznidazole or nifurtimox and subsequent recovery in all patients that received chemotherapy.

The existence of differences in susceptibility to these compounds of a large number of *T. cruzi* isolates from different host, and geographic areas have been reported [8, 20], suggesting that TcI epimastigotes are more resistant to antichagasic drugs than other DTUs [8, 62]; however other surveys did not find differences in drug susceptibility in human chronic cases infected with TcI [21, 22]. Although in our study all clones belong to TcI, we found that Girón clones that displayed higher sensitivity to nifurtimox presented a particular G2 genotype and C/T transition in *Cyt b*. Studies with human isolates obtained in acute phase of CD in Brazilian Amazonia did not find differences in susceptibility to the drug between different DTUs [63]. However, they found statistically significant differences between TcI isolates from different regions of the Amazonia, similar results to those observed by us with the Girón outbreak clones. Similarly, in Venezuela outbreaks of oral acute CD, the major failure of the treatment was observed in patients infected with TcId (G11) showing greater susceptibility of the genotypes TcIb (G2) [34]. Alike, in Mexican isolates from acute and chronic cases found differences in susceptibility between different genotypes of TcI [64]. Further studies with these isolates could elucidate whether there are genotypic differences responsible for these resistance phenotypes, and thus achieve greater understanding of drug resistance mechanisms. In this study, when the diagnosis was appropriate and early, all treated cases responded well to antichagasic treatment, which highlights the importance of diagnosis and treatment early to prevent fatal outcomes associated with these acute episodes.

## Conclusions

To summarize, all *T. cruzi* clones belong to DTU I and two intra-DTU genotypes were found with the sequencing of the mini-exon intergenic spacer, however there is no strict correlation between genetic groups, and the cycles of the parasite or the clinical forms of the disease. The cardiac tissue showed intense inflammatory infiltrate, myocardial necrosis and abundant amastigote nests. However, although the gastrointestinal tissue was congestive, no inflammation or parasites were observed. These findings represent a major contribution to studies aiming to associate the etiologic agent features with host behavior, and highlight the complexity of identifying characteristics of the parasite that could be involved in the pathogenesis of the acute Chagas disease.

## Additional file

Additional file 1: Figure S1. Genotyping of *T. cruzi* isolates. Mini-exon intergenic spacer, 24Sα rRNA, 18S rRNA, glucose-6 phosphate isomerase (GPI), heat shock protein 60 (HSP60), and mitochondrial gene Cytochrome Oxidase subunit II (COII) genes were characterized. (TIF 386 kb)
