# Supplementary figures and images for: Acute chagas outbreaks: molecular and biological features of Trypanosoma cruzi isolates, and clinical aspects of acute cases in Santander, Colombia
Source: Parasit Vectors. 2015 Nov 26;8:608. doi: 10.1186/s13071-015-1218-2 (PMC4661967; doi:10.1186/s13071-015-1218-2)

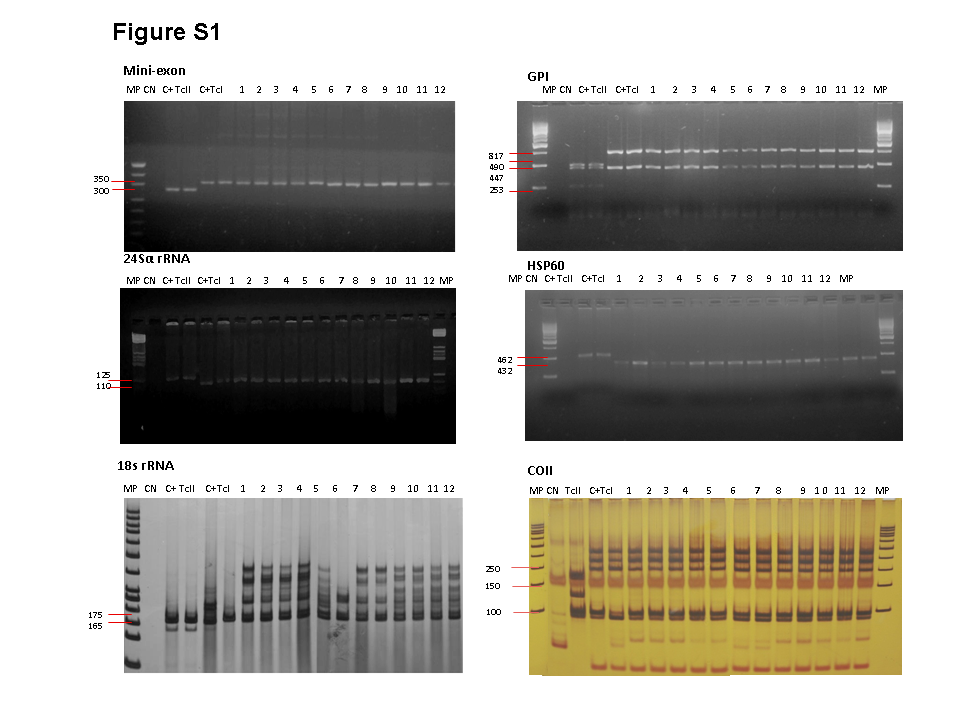

Supplement: Additional file 1: Figure S1. — Genotyping of T. cruzi isolates. Mini-exon intergenic spacer, 24Sα rRNA, 18S rRNA, glucose-6 phosphate isomerase (GPI), heat shock protein 60 (HSP60), and mitochondrial gene Cytochrome Oxidase subunit II (COII) genes were characterized. (TIF 386 kb) [file 13071_2015_1218_MOESM1_ESM.tif]
